# lncRNAs as prognostic markers and therapeutic targets in cuproptosis-mediated cancer

**DOI:** 10.1007/s10238-024-01491-0

**Published:** 2024-09-26

**Authors:** Asif Ahmad Bhat, Muhammad Afzal, Ehssan Moglad, Riya Thapa, Haider Ali, Waleed Hassan Almalki, Imran Kazmi, Sami I. Alzarea, Gaurav Gupta, Vetriselvan Subramaniyan

**Affiliations:** 1 Uttaranchal Institute of Pharmaceutical Sciences, Uttaranchal University, Dehradun, India; 2 Department of Pharmaceutical Sciences, Pharmacy Program, Batterjee Medical College, P.O. Box 6231, 21442 Jeddah, Saudi Arabia; 3 Department of Pharmaceutics, College of Pharmacy, Prince Sattam Bin Abdulaziz University, 11942 Al Kharj, Saudi Arabia; 4 Centre for Global Health Research, Saveetha Medical College, Saveetha Institute of Medical and Technical Sciences, Saveetha University, Chennai, India; 5 Department of Pharmacology, Kyrgyz State Medical College, Bishkek, Kyrgyzstan; 6 Department of Pharmacology, College of Pharmacy, Umm Al-Qura University, Makkah, Saudi Arabia; 7 Department of Biochemistry, Faculty of Science, King Abdulaziz University, 21589 Jeddah, Saudi Arabia; 8 Department of Pharmacology, College of Pharmacy, Jouf University, 72341 Sakaka, Aljouf Saudi Arabia; 9 Centre for Research Impact & Outcome, Chitkara College of Pharmacy, Chitkara University, Rajpura, Punjab, 140401 India; 10 Centre of Medical and Bio-Allied Health Sciences Research, Ajman University, Ajman, United Arab Emirates; 11 Pharmacology Unit, Jeffrey Cheah School of Medicine and Health Sciences, Monash University Malaysia, Jalan Lagoon Selatan, 47500 Bandar Sunway, Selangor Darul Ehsan Malaysia; 12 Department of Medical Sciences, School of Medical and Life Sciences, Sunway University, Bandar Sunway, 47500 Subang Jaya, Selangor Malaysia

**Keywords:** Long non-coding RNAs, Cuproptosis, Cancer, Cellular process, Lung

## Abstract

Long non-coding RNAs (lncRNAs) have emerged as crucial regulators in various cellular processes, including cancer progression and stress response. Recent studies have demonstrated that copper accumulation induces a unique form of cell death known as cuproptosis, with lncRNAs playing a key role in regulating cuproptosis-associated pathways. These lncRNAs may trigger cell-specific responses to copper stress, presenting new opportunities as prognostic markers and therapeutic targets. This paper delves into the role of lncRNAs in cuproptosis-mediated cancer, underscoring their potential as biomarkers and targets for innovative therapeutic strategies. A thorough review of scientific literature was conducted, utilizing databases such as PubMed, Google Scholar, and ScienceDirect, with search terms like 'lncRNAs,' 'cuproptosis,' and 'cancer.' Studies were selected based on their relevance to lncRNA regulation of cuproptosis pathways and their implications for cancer prognosis and treatment. The review highlights the significant contribution of lncRNAs in regulating cuproptosis-related genes and pathways, impacting copper metabolism, mitochondrial stress responses, and apoptotic signaling. Specific lncRNAs are potential prognostic markers in breast, lung, liver, ovarian, pancreatic, and gastric cancers. The objective of this article is to explore the role of lncRNAs as potential prognostic markers and therapeutic targets in cancers mediated by cuproptosis.

## Introduction

LncRNAs are emerging as pivotal regulators in cellular biology despite their lack of protein-coding potential [1]. These RNA molecules, typically longer than 200 nucleotides, have garnered significant attention due to their diverse roles in critical cellular processes such as gene expression regulation, chromatin remodeling, and cell differentiation [2]. By interacting with various molecules within the cell, lncRNAs can modulate gene expression at multiple levels, influencing mRNA stability, splicing, and even serving as endogenous RNAs (ceRNAs) to sequester microRNAs (miRNAs) [2]. Such functions have made lncRNAs central players in the development and progression of numerous diseases, including cancer, where specific lncRNAs like HOTAIR, MALAT1, and HOTAIR have been linked to metastasis, highlighting their potential as therapeutic targets and biomarkers [3].

In parallel with the growing understanding of lncRNAs, a novel form of programmed cell death, termed cuproptosis, has been discovered [4]. Unlike traditional cell death mechanisms such as apoptosis and necrosis, cuproptosis is triggered by the toxic accumulation of copper within cells [5]. This excess copper disrupts mitochondrial function, particularly impacting proteins involved in the Krebs cycle, leading to an overproduction of reactive oxygen species (ROS). The resulting oxidative stress induces cell death, marking cuproptosis as a unique and distinct pathway in cellular degradation [6].

The intersection of lncRNAs and cuproptosis presents a promising new avenue in cancer research. Tumor cells often exhibit dysregulated copper metabolism, leading to elevated copper levels that make them particularly susceptible to cuproptosis [7]. LncRNAs, with their ability to influence gene expression and cellular responses, are now being studied for their role in regulating cuproptosis-related pathways [8]. For example, lncRNAs like MALAT1 and HOTAIR have been found to interact with mitochondrial components and copper transporters, thereby modulating the sensitivity of cancer cells to cuproptosis [4]. This interaction suggests that lncRNAs could serve not only as biomarkers for cancer prognosis but also as potential therapeutic targets to induce selective cell death in tumors [9]. The potential of cuproptosis in cancer therapy lies in its ability to target cancer cells with minimal damage to normal tissues selectively. By leveraging the dysregulation of copper metabolism in tumor cells, researchers are exploring ways to harness cuproptosis as a novel treatment strategy [10].

Furthermore, the role of lncRNAs in this process adds another layer of complexity and opportunity, as these molecules could be manipulated to enhance the efficacy of cuproptosis-based therapies [3]. The purpose of this review is to explore and elucidate the emerging roles of lncRNAs in the regulation of cuproptosis, a novel form of programmed cell death driven by copper accumulation. By examining the intricate interactions between lncRNAs and cuproptosis-related pathways, this review aims to provide new insights into the molecular mechanisms underlying copper-induced cell death and its implications for cancer therapy.

## Long non-coding RNAs: An overview

### Biogenesis and classification

LncRNAs are transcribed from DNA by RNA polymerase II and processed through many post-transcriptional modifications that messenger RNAs go through, like 5' capping, poly-adenylation, or splicing [11]. Nonetheless, they also exhibit unique splicing and are less restricted than their protein-coding counterparts [12]. Biogenesis of lncRNAs involves transcription from different genomic locations and processing events that can affect their stability, localization, and function [13]. It is classified into lncRNAs depending on their location and functions within the genome [14]. Intergenic lncRNAs are transcribed in intergenic regions between coding genes and participate in further gene regulation chromatin dynamics [15]. The intronic lncRNAs are derived from their presence within introns in protein-coding genes, which regulate their host genes' splicing and gene expression levels [16]. LncRNAs transcribed from promoters that control gene activity are known as promoter-associated lncRNA [17]. LincRNAs (long intergenic non-coding RNAs) sometimes regulate gene expression over long genomic distances [18]. Antisense lncRNAs are divergently derived from the opposite strand of coding genes and work to regulate gene expression by interacting with RNA or chromatin [19]. The complexity of lncRNA function can be partially attributed to the specificity shown by each class to mediate cellular processes and gene regulation, thereby preserving normality within a cell or tissue while also contributing to the establishment of pathology.

### Functions in cellular processes

**Fig. 1:**
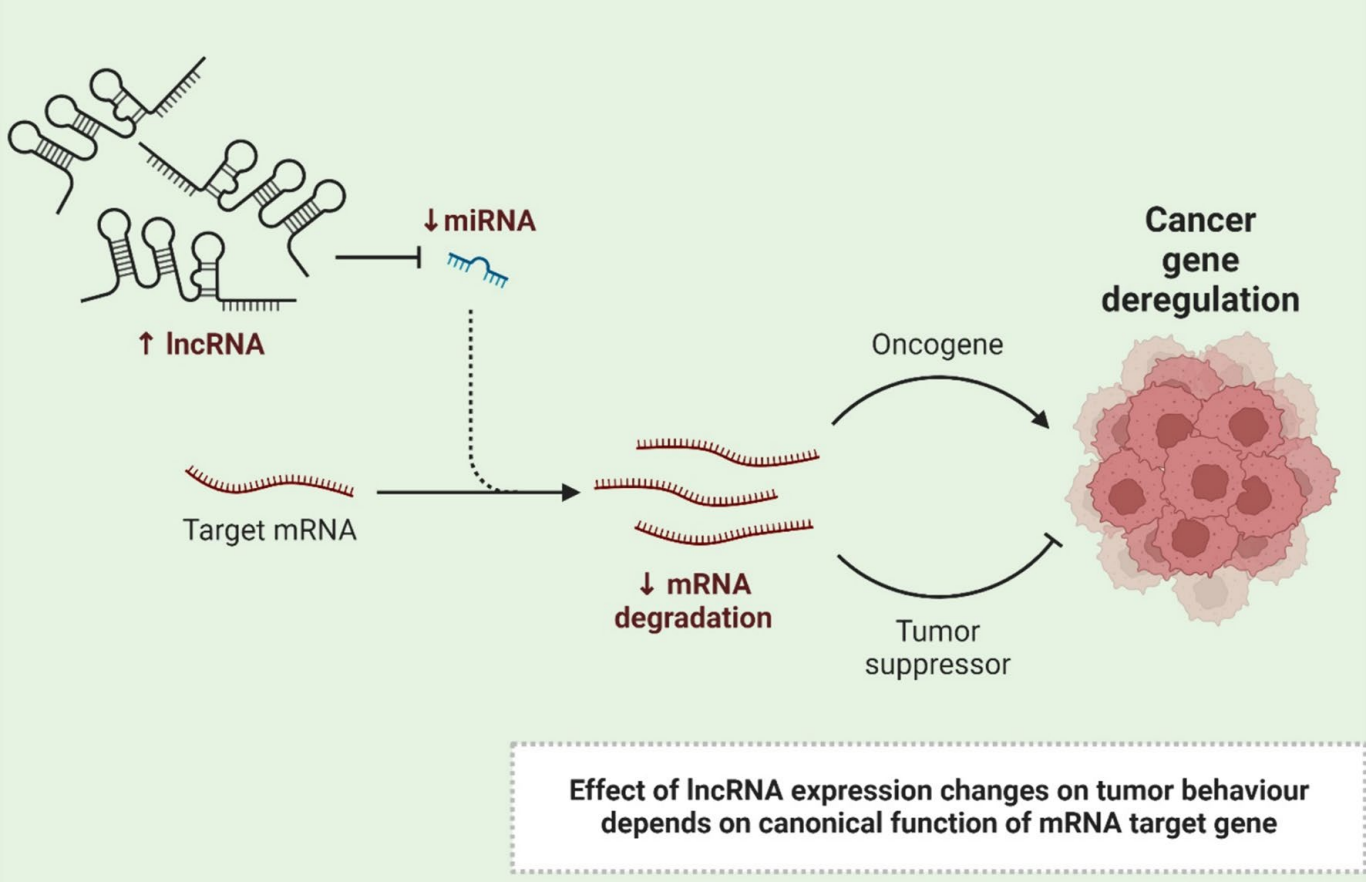
The figure illustrates how increased lncRNA expression decreases miRNA levels, reducing target mRNA degradation, leading to oncogene activation or tumor suppressor inhibition, ultimately causing cancer gene deregulation

LncRNAs play a central role in modulating many cellular processes by interacting with proteins, DNA, and other classes of RNA transcripts [1]. Through the recruitment of chromatin-modifying complexes, they exert widespread control over gene expression [20]. LncRNAs bring histone modifiers to promoters or enhancers and change chromatin structure, affecting the transcription of neighbor genes [21]. LncRNAs also participate in transcriptional control by being enhancers or repressors, with different mechanisms on transcriptions' initiation and elongation phases [22]. They regulate the expression of target genes at various levels post-transcriptionally by tweaking mRNA splicing, stability, and translation [23]. The lncRNAs can bind to the mRNA, altering alternative splicing events and replacing somatic hypermutation components like stem-loops or activating factors, modulating mRNA stability [24]. In addition to these functions, lncRNAs are also extremely important for cellular signaling, as they can scaffold signaling complexes or modulate the activity of different signaling proteins [25]. They are also required for proper cellular differentiation and development, controlling the expression of pivotal developmental genes and orchestrating transitions between cell fates [26]. In addition, lncRNAs mediate cellular stress responses by controlling key elements of the transcriptional and post-transcriptional machinery that govern expression levels of genes involved in apoptosis, survival or autophagy [27] (Fig. 1).

## Cuproptosis: A novel cell death pathway

### Mechanism of cuproptosis

The accumulation of copper ions through its direct interaction with mitochondrial components disturbs cellular homeostasis, leading to this particular mechanism of cell death [28]. The start of cuproptosis involves the accumulation of excess copper ions in a cell [29]. Copper is a fundamental micro-element that becomes toxic when it exceeds physiological limits [30]. The surplus copper is associated with lipoylated forms of tricarboxylic acid (TCA) pathway intermediates, a key mitochondrial energy production conduit [31]. For example, copper intercalates with lipoylated proteins in the TCA cycle, turning over and making these forms more unstable [32]. This defect leads to a major metabolic function of causative mitochondrial activity and excessive ROS production [33]. Oxidative stress produces mitochondria dysfunction, leading to cell death [34]. Moreover, the raised ROS per se damages cellular content and activates further cell stress/ damage response, accelerating apoptosis in a feed-forward manner [35]. Cuproptosis is not dependent on caspase activation nor shares the morphological features of apoptosis [36]. Instead, this results from copper's direct effects on mitochondrial proteins [37].

According to recent research, lncRNAs could be essential in controlling important cuproptosis pathway elements. The expression of copper transporters like CTR1 and ATP7A/B, which are essential for preserving intracellular copper levels, may be influenced by these lncRNAs [38]. When these transporters are dysregulated, copper may build up, which can cause mitochondrial malfunction and, eventually, cell death. Furthermore, lncRNAs may regulate genes related to mitochondrial respiration and the generation of ROS, which helps to fine-tune the mitochondrial responses to copper stress [39]. This control is essential because high levels of ROS from mitochondria may worsen cellular damage and trigger cuproptosis. Furthermore, under circumstances of copper toxicity, lncRNAs may interact with proteins directly involved in the cuproptosis machinery, affecting their stability or activity [40].

### Differences from other cell death pathways

Cuproptosis is characterized by the retention of copper inside a cell that leads to apoptosis [41]. It significantly differs from other cell-dying methods, including apoptosis, necrosis, autophagy, pyroptosis, and ferroptosis [42, 43]. Apoptosis is initiated when a cell is damaged and results in a stream of intracellular response, which includes a series of events, such as activation of caspase and DNA fragmentation, causing cell death [44]. As for necrosis, it is elicited by acute injury and immediately causes cytoplasm retention and cell lysing [45]. However, cuproptosis is different since it involves more regulated and controlled methods of cuproptosis inside a cell [46]. Also, autophagy is a survival mechanism developed by a cell under stress, and it includes organelles digestion and recycling [47]. Thus, again, cuproptosis is different because it is related to the toxicity of copper inside a cell [48]. Finally, pyroptosis is initiated by inflammasome activation and gasdermins pore development [49]. Ferroptosis is related to iron lethality, but only in the case of cuproptosis, death is caused by the lethal amount [50].

## Cuproptosis in *cancer*

### Role of cuproptosis in *cancer* development and progression

Copper is a critical trace element that plays a crucial role in angiogenesis, oxidative stress response, enzyme function, and electron transfer [51]. Copper homeostasis is altered in cancer cells, significantly increasing copper levels [52]. It has also been shown that the role of cancer cell growth, metastasis, angiogenesis, and copper is directly related [53]. Therefore, the trace element may be considered essential in the survival and proliferation of cancer cells [54]. In this new model, cuproptosis, copper accumulates in the mitochondria, leading to protein aggregation and the formation of toxic protein levels [55]. This culminates in the loss of the mitochondrial potential function and cell death [56]. Cu is essential for cell metabolism and, simultaneously, deadly to the cells when present in either low or high amounts [57]. There are two ways to achieve cuproptosis: modulating the copper levels themselves or disrupting the copper-binding proteins [58]. Combining cancer therapies with these bronze-era chemicals is likely better than the use of singular [59]. Combining the two compounds increases the effectiveness of the therapeutic actions, consequently reducing the side effects [60].

### Cuproptosis dysregulation in different *cancer* types

Cuproptosis, copper-induced cell death via mitochondrial dysfunction, results from specific copper metabolism dysregulations responsible for copper’s roles across different cancer types [61]. In breast cancer, abnormally elevated copper levels stimulate angiogenesis and metastasis and can be targeted effectively by cuproptosis [62]. The high intracellular copper concentration of lung cancer cells implies that cuproptosis-inducing agents could be used effectively against those cells [63]. Hepatocellular carcinoma exhibits some of the most substantial copper dysregulation, which might be related to the function of the liver in copper metabolism [64]. As a result, liver cancer might stand to benefit the most from this novel therapeutic avenue, especially in the advanced stable phase [65]. Colorectal cancer cells often have dysregulated copper metabolism, which means altering the copper levels could lead to cell death and possibly more effective inhibition of the tumor progression overall [8]. Pancreatic cancer is currently complicated to treat, but copper dysregulation offers another potential therapeutic use of cuproptosis [8]. Finally, increased copper amounts are also linked to prostate cancer’s progression, which, in the case of castration-resistant prostate cancer, could lead to potentially effective new treatments [8].

## LncRNAs in cuproptosis regulation

### LncRNAs influencing cuproptosis-related genes

LncRNAs control cuproptosis-related genes, which alters their impact on cells when exposed to copper-induced stress [66]. Mitochondria become dysfunctional and become the primary target for copper, resulting in cuproptosis [67]. LncRNA alters this stress's effects by controlling the expression of beneficial and necessary genes responsible for mitochondria’s correct function [68]. It can also change the concentration of intracellular transporters, such as CTR1 and ATP7A, which support the error of copper into and from the cell [69]. It means that it controls the amount of copper used and accumulated in mitochondria. Copper transport ATPases ATP7A and ATP7B are essential for the export and distribution of copper [70]. At the same time, lncRNAs also impact mitochondrial stress response and apoptotic pathways [71]. This occurs as the lncRNAs control the expression of genes, which mitochondria damage may lead to cell death [72]. In detail, mitochondria experience excessive stress and instigate the early stage of the apoptosis process [73]. Some lncRNAs can increase the genes that protect mitochondria from oxidation and free radicals or promote the creation of apoptosomes that cause cell death [74]. Scientists can create methods of targeting specific lncRNAs that control copper metabolism and mitochondria’s response to eliminate cuproptosis [75]. It would improve the effectiveness of treatment and decrease the chance of getting resistance [76].

The functional variety of lncRNAs makes it difficult to pinpoint their precise involvement in cuproptosis and to comprehend how they vary throughout cancer types. The lncRNA MALAT1 has been shown to be involved in a number of cellular functions, such as cell cycle control and metastasis [77]. More recently, research has indicated that it may affect cuproptosis by modifying mitochondrial activity and oxidative stress responses [78]. On the other hand, it seems that MALAT1's effects on cuproptosis varied depending on the cancer cell line. MALAT1 may have a protective effect by maintaining mitochondrial membranes in some malignancies. Still, it may also promote copper-induced cell death in other cancers by upregulating genes implicated in mitochondrial ROS generation [79].

In addition to its well-known functions in chromatin remodeling and gene silencing, HOTAIR is another lncRNA that may interact with pathways linked to cuproptosis, according to new research. Specifically, HOTAIR has been linked to the control of CTR1, a copper transporter that is essential for preserving intracellular copper concentrations. HOTAIR may alter cancer cells' sensitivity to cuproptosis by affecting CTR1 expression; however, the precise processes behind this effect are yet unknown and probably varied throughout cancer types [80].

### Interaction between lncRNAs and cuproptosis pathways

The interaction of lncRNAs and cuproptosis pathways is an emerging study area with substantial implications for understanding and controlling cell responses to copper-induced stress [81]. Cuproptosis is a distinct form of cell death caused by copper accumulation, which disrupts mitochondria function and damages the cell [82, 83]. LncRNAs are central to adjusting the process and controlling the action of essential components of copper metabolism and mitochondrial function [84]. They target the copper transporter ATP7A, an integral component of the cellular machinery that manages copper levels [85]. Quantitative polymerase chain reaction studies have determined that modulation of CSNK1D by the lncRNA and other genes plays a vital role in cuproptosis [86]. The mitochondria are central to the process and are linked to the calculated signaling pathways of apoptosis, which is responsible for cuproptosis [87]. The novel interactions have substantial implications for cancer. In the context of loss of heterozygosity, cancer cells tend to turn into heterozygous ones and begin exhibiting sensitivity to copper-induced stress and death linked to cuproptosis [88]. The lncRNAs may serve as the primary target in the process associated with cancer and contribute to the anticipated outcomes, which offer insights into desirable treatment regimens that trigger cuproptosis [89].

## Interplay of lncRNAs with key signaling pathways in *cancer* and cuproptosis

The ability of lncRNAs to interact with multiple signaling pathways makes them crucial players in modulating various aspects of tumor biology. The PI3K/AKT/mTOR signaling pathway is a key player in controlling metabolism, cell division, and survival. It has been shown to interact with a number of lncRNAs, affecting how they function in cuproptosis and the development of cancer [90]. It has been shown that the lncRNA H19 modulates the PI3K/AKT pathway by sponging miR-29a and serving as a ceRNA, thereby upregulating PI3K expression. In certain cancer types, this interaction may be able to offset the effects of cuproptosis by improving cell survival and proliferation [91]. Targeting this lncRNA may interfere with the PI3K/AKT/mTOR pathway in malignancies that overexpress H19, making cells more susceptible to copper-induced apoptosis.

Another important signaling cascade involved in cell migration, differentiation, and proliferation is the Wnt/β-catenin pathway [92]. It has been shown that the MALAT1 interacts with this pathway, facilitating the spread of cancer and increasing apoptotic resistance. MALAT1 can control β-catenin expression, which improves the transcription of Wnt target genes that encourage the development of tumors [93]. MALAT1 may shield cancer cells from copper-induced stress in the setting of cuproptosis by maintaining Wnt/β-catenin signaling, which promotes cell survival and proliferation. When Wnt signaling is active in malignancies, inhibiting MALAT1 may interfere with this protective function and increase the cells' susceptibility to cuproptosis [94].

In response to cellular stress, the tumor suppressor protein p53 is essential for controlling the cell cycle and programmed cell death [95]. Numerous lncRNAs have been linked to the regulation of the p53 pathway, such as PVT1 and NEAT1. PVT1 has the potential to influence the stability and transcriptional activity of p53, while NEAT1 is known to control p53-dependent apoptosis. LncRNAs such as PVT1 may affect the ratio of apoptosis to cuproptosis in tumors with p53 pathway mutations [96]. Overexpression of PVT1 may stabilize mutant p53 and allow cells to survive stress caused by copper. Autophagy is a cellular mechanism that facilitates the destruction and recycling of damaged proteins and organelles. It is also a route that interacts with the activity of lncRNA [27]. It has been shown that the lncRNA HOTAIR inhibits autophagy by downregulating the expression of genes linked to autophagy, including ATG5 and ATG7 [97]. In the case of copper stress, HOTAIR may worsen mitochondrial dysfunction and encourage cuproptosis by blocking autophagy, thus impeding the removal of damaged mitochondria [98].

## LncRNAs in cuproptosis-mediated *cancer*

### LncRNAs in cuproptosis-mediated breast *cancer*

Breast cancer is a condition concerning a large number of the population. It is one of the most commonly occurring cancers among women all over the world, with the chance of being diagnosed in men also [99]. Breast cancer is of various types, showing different manifestations, characteristics, and therapies, and has other implications for the prognosis [100]. Alongside cancers of other body organs, breast cancer has several subtypes, each having distinct characteristics [101]. The types of breast cancer discussed here are invasive ductal carcinoma and invasive lobular carcinoma, in which both types are invasive [102]. Breast cancer also has a noninvasive subtype known as ductal carcinoma in situ, which is confined in the ducts and, therefore, has a better prognosis with little chance of being life-threatening [103]. Triple-negative breast cancer is a subtype in which it is not positive for estrogen, progesterone, and HER, the three commonly occurring receptors in any breast cancer type [104]. NOMA is a subtype having the highest overexpression and growth rate [105]. Chemosensitivity is the susceptibility of cancer cells to chemotherapy agents [106]. Chemosensitivity is responsible for the treatment mechanism through personalized therapy [107]. A chemosensitivity test is an assay developed by the oncologist for their better understanding and indication or confirmation of the susceptibility or resistance or the testing conditions of chemotherapeutic agents developed [108]. Thus, chemosensitivity is the main or the first and foremost factor directing oncologists in identifying the drugs to which the cancer is susceptible [109]. The other factors determining the same factor are the genetic mutation or the tumor microenvironment of that particular person [110]. Wu et al. identified SLC31A1 as a cuproptosis-related gene in breast cancer. SLC31A1 in the breast cancer tissues was upregulated and indicated poor prognosis, immune cell infiltration, and chemosensitivity. The LINC01614/miR-204-5p/SLC31A1 axis may play a major role in cuproptosis-related breast cancer [111]. Therapy sensitivity means the patient's response to the treatment, whether drugs, psychotherapy, or other therapies [112]. It varies because of individuals and the disease’s nature [113]. The sensitivity can be assessed by symptom changes and biomarkers [114]. It is beneficial to understand the therapy of patients to individualize it, increasing the chances of recovery and success [115]. In this article, Wu et al. investigated the cuproptosis-related lncRNAs in breast cancer, showing how they are associated with prognosis and therapy sensitivity. They identified six lncRNAs, building a model that classified the patients in terms of risk groups. This model could be potentially used to predict the response to the therapy and the chances of survival [116].

**Fig. 2:**
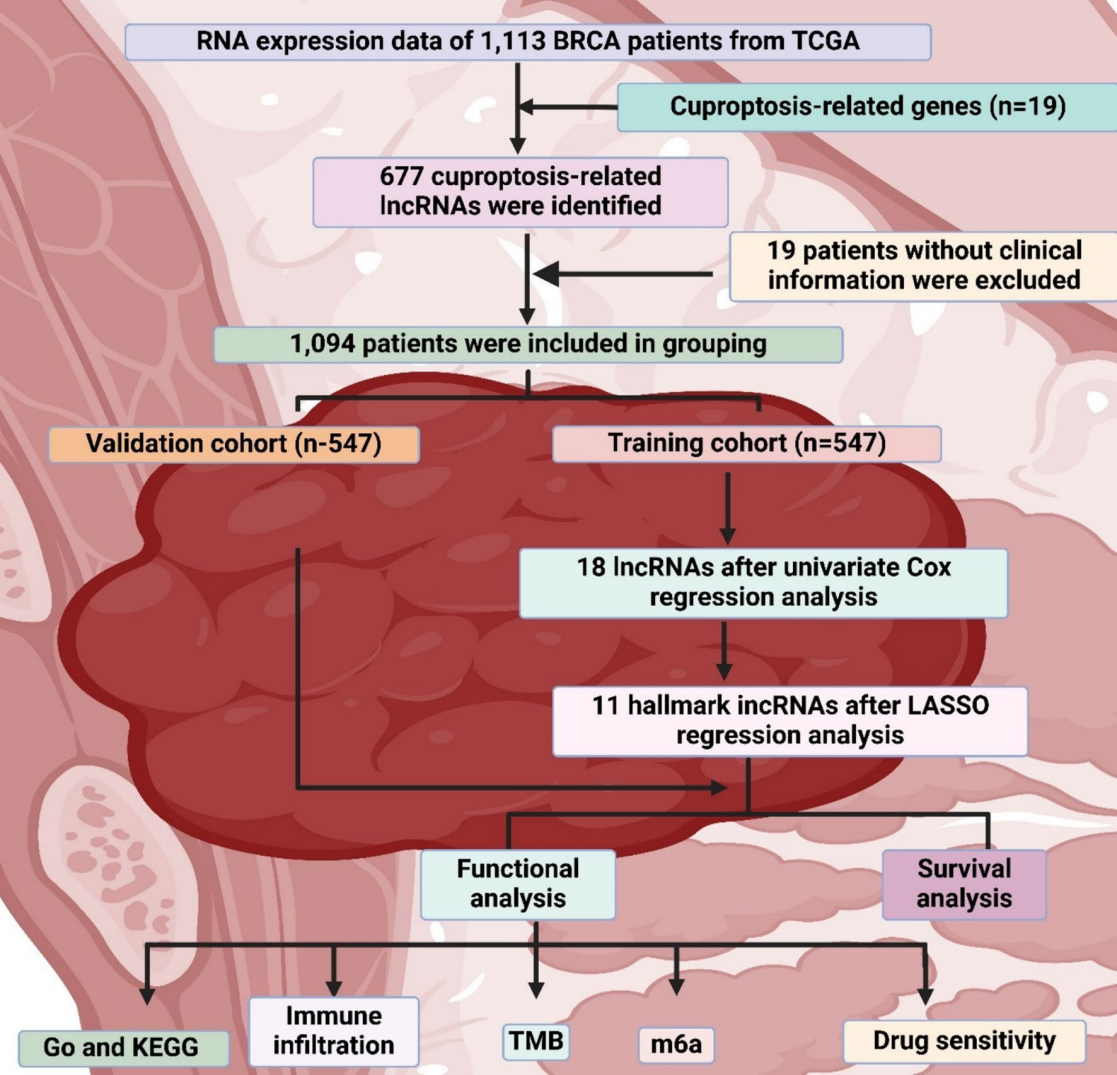
The figure outlines the identification and analysis of cuproptosis-related lncRNAs in BRCA patients, highlighting 11 hallmark lncRNAs for functional and survival analysis, including GO/KEGG, immune infiltration, TMB, m6A, and drug sensitivity

The immune microenvironment is a network of immune cells, stromal components, and signaling molecules around a tissue or tumor [117]. It controls immune response and affects disease development and response to therapy [118]. Understanding this microenvironment is vital for immunotherapy development and predicting the course of various diseases' responses to treatment [119]. Jiang et al. created a predictive model using 11 cuproptosis-related lncRNAs to determine breast cancer prognosis and immune microenvironment. In patients with high-risk, overall survival was lower and had different mutation profiles [120]. A similar study by Xu et al. developed a 2-lncRNA signature for breast cancer prognosis related to cuproptosis. The signature, BCCuS, was validated as a prognostic factor and could help predict therapy response and patient survival [121]. In another study, Pan et al. investigated cuproptosis-related lncRNAs for their prognostic role and effect on the immune microenvironment for breast cancer. The identified model based on ten lncRNAs could predict survival and drug sensitivity [122]. The immune checkpoint is an essential regulator of the immune system, which ensures that immune responses are proportionate, contain no autoimmunity, and that tissue damage during the reaction is minimized [123]. It is molecules on immune cells, especially T cells, that must be activated to start an immune response [124]. Although the immune checkpoint serves as a protection layer, it is activated by cancer cells to escape detection and elimination by the immune system [125]. Yu et al. established a predictive risk score system for breast cancer, which is based on 11 cuproptosis-related lncRNAs. This system can predict patients’ prognostic outcomes, immune status, and drug sensitivity to aid their treatment, as shown in Fig. 2 [126].

Jiang et al. described a target signature for cuproptosis-associated results and axon expression in breast cancer. lncRNAs correlated with the prognosis were detected in database mining analysis [127]. The study described the lncRNAs related to the prognosis. Another study by Li et al. analyzed the role of cuproptosis-related lncRNAs using prognosis prediction and immune infiltration in breast cancer. A predictive model was developed based on the lncRNA expression levels, and the signature was associated with specific outcomes and responses to therapeutics [116] (Fig. 2).

### LncRNAs in cuproptosis-mediated lung *cancer*

Lung cancer is a widespread type of cancer that remains highly lethal [128]. It occurs in the tissues of the lungs, commonly within the cells lining the air passages [129]. Lung cancer can be divided into two categories, NSCLC and SCLC, which differ in unique characteristics, influence factors, and therapy methods and outcomes [130]. About 85% of all cases are non-small cell lung cancer, which has several subtypes [131]. Squamous cell carcinoma is typically found within the central aspect of the lungs and is closely attached to smoking [132]. Large cell carcinoma does not apply to the other categories but can arise in any lung part; it generally grows rapidly and moves quickly to develop, which complicates the treatment process [133]. Small cell lung cancer accounts for nearly 15% of all cases [134]. It is strongly connected with smoking and demonstrates itself through its dominating cytologic profile, extreme acting, and speedy growth [135]. Generally, it begins in the central airways and quickly shifts to other body parts, such as the liver, bones, and brain [136]. Fast growth and high aggression suggest that it has already reached a practically developed level at a stage where it is detectable, which complicates the treatment and the following survival rates [137]. Wang et al. studied the cuproptosis-related lncRNAs in lung adenocarcinoma. They used the TCGA and GEO data to detect cuproptosis-related lncRNAs and detect 16 candidates before developing the prognostic signature. They found that the identified long non-coding RNAs are associated with poor overall survival and progression-free survival. They can predict prognosis and immune escape, indicating potential clinical applications and improvements [56].

**Fig. 3:**
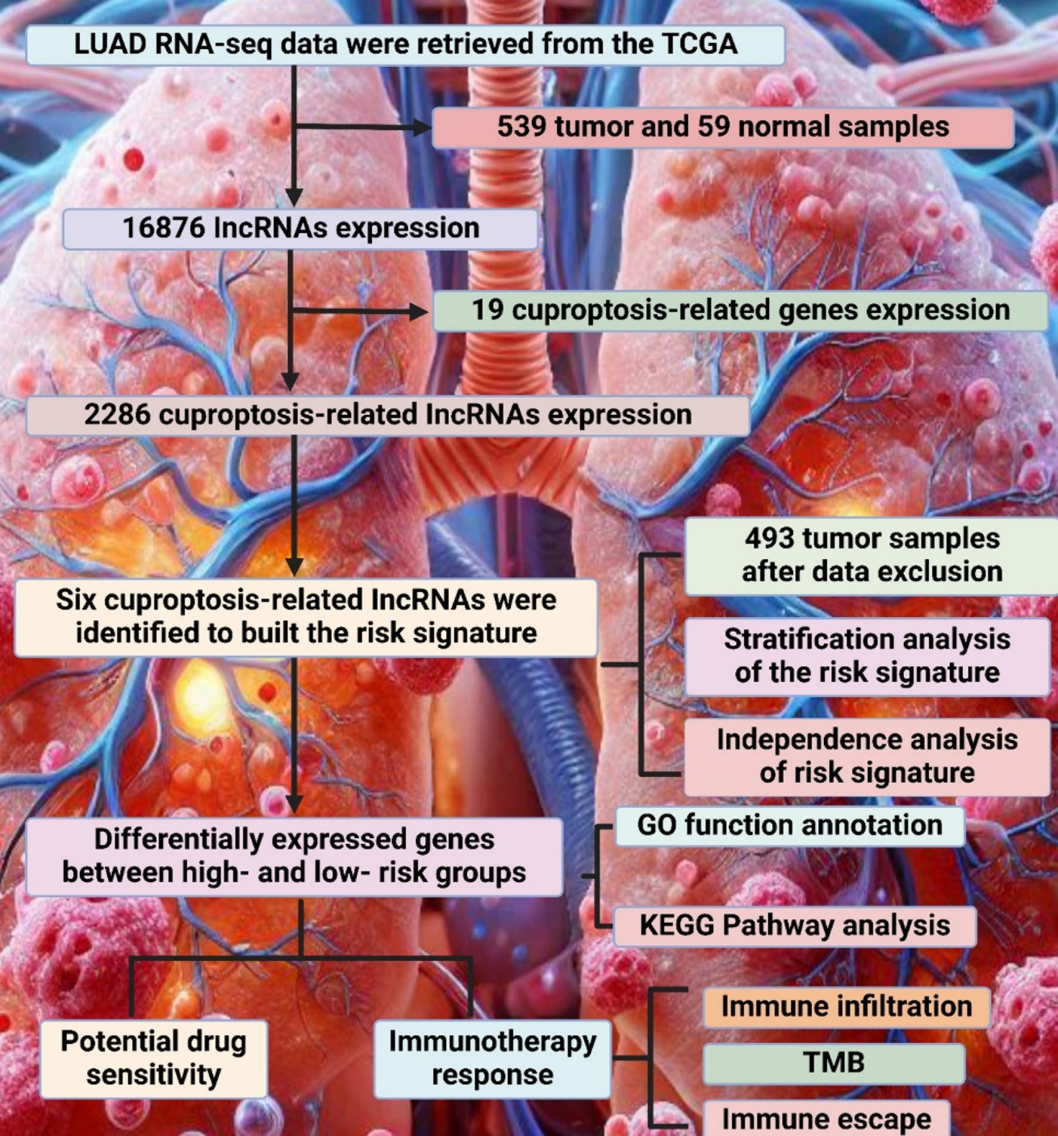
The figure shows the identification of six cuproptosis-related lncRNAs in LUAD from TCGA data, analyzed for risk signature, gene expression, drug sensitivity, immunotherapy response, GO, KEGG, TMB, and immune infiltration

Radiosensitization-related cuproptosis is a novel concept in the cancer treatment context, which combines the principles of radiosensitization and the recently discovered mechanism of cell death, cuproptosis [138]. Radiosensitization implies making a cancer cell more sensitive to the impacts of radiation therapy and, therefore, improving the effect of the treatment [139]. Cuproptosis is a type of programmed cell death, which is driven by copper ions serving to trigger the toxicity of a cancer cell [140]. Additionally, Xu et al. developed a cuproptosis-related lncRNA signature to estimate the prognosis of NSCLC patients after applying radiation therapy. The team constructed a prediction model of six lncRNAs and used it in training and testing groups. The results proved its accuracy in predicting patient prognosis more productively than traditional clinical factors [141]. According to another study by Yu et al., a cuproptosis-related lncRNA signature can predict prognosis and immunotherapy responses in LUAD patients. The authors identified six prognosis-associated key lncRNAs and employed numerous analytical approaches to validate their predictive values. Overall, the study demonstrated that patients at high risk often exhibit poorer survival rates; in addition, this research suggested some therapeutic options based on the analyzed lncRNA profile, as shown in Fig. 3 [142].

GSVA and GSEA are computational methods for interpreting gene expression profiles [143]. GSVA evaluates variation in pathway activity across samples, while GSEA identifies statistically significant gene sets enriched in different phenotypes [144]. Wang et al. constructed a novel ten-cuproptosis-related lncRNAs-based prognostic signature for LUAD. GSVA and GSEA, among other analyses, were used to evaluate the impact of the lncRNAs on prognosis and the immune landscape. Their findings showed that the signature could predict patient survival and guide therapeutics, including drug sensitivity [145]. Tumor cell proliferation is one of the most prominent characteristics of cancer, referring to the unrestricted and abnormally high reproduction of these cells [146]. This results from genetic aberrations as the unstable cell cycle continues due to mutations [147]. Importantly, this activity is vital for the growth, metastasis, and development of cancer, making it one of the most researched aspects of these conditions [148, 149]. Sun et al. presented a novel tested signature composed of six lncRNAs capable of reliably predicting the patient’s condition and guiding the optimum course of treatment. Additionally, their subsequent confirmation experiments uncovered that one of these cancers-suppressing lncRNAs, NIFK-AS1, could arrest the proliferation and migration of tumor cells [150].

Immune function is assessed to diagnose, monitor, and treat numerous medical conditions, including infections, autoimmune diseases, immunodeficiencies, and cancers [151]. Both clinical evaluation and laboratory tests are crucial components of comprehensive assessment [152]. Medical history and physical examination may reveal signs of recurrent infections, autoimmune symptoms, or a family history of immune disorders [153]. Physical examination may also show signs of acute or chronic infection, inflammation, or immunodeficiency[154]. According to the study by Zhao et al., their five-cuproptosis-related lncRNA signature for the prediction of prognosis, immune function, and drug sensitivity in LUSC clearly distinguished high- and low-risk patients and armed other specialists with considerable information also regarding immune function and drug response. The results showed differences in immune pathways and suggested potential therapies based on risk scores [150]. Copper homeostasis refers to the conjunction of processes to maintain optimal copper levels in the human body [155]. Copper is a vital trace element that performs a variety of functions in the human system, such as providing integrity while partaking in the DNA repair mechanism, vitalization of enzymes, forming strong ties between collagens and elastins in connective tissues, relaying chemical signals through the nervous system, and moisture distribution throughout the body [156]. At the same time, both copper deficiency and overload can induce devastating health effects, thus making it vital for the element’s amounts to be guarded cautiously [157]. Ma et al. considered the connection between copper homeostasis, cuproptosis, and lncRNA in LUAD. They used the public database TCGA and GEO and created a prognostic lncRNA signature, which can be vital for understanding the patients’ prognosis and available therapy methods. The authors studied the impact of this process in cancers focusing on individualized immunotherapy [158] (Fig. 3).

### LncRNAs in cuproptosis-mediated ovarian *cancer*

Ovarian cancer is one of the most severe cases of gynecologic cancers due to a high degree of late diagnosis and aggressive growth of the tumor [159]. Depending on the type of cells from which cancerous formation emanates, it is generally accepted to distinguish three main types of the disease: epithelial tumors, germinative tumors, and tumors of the ovarian stroma [160]. Epithelial tumors form on the ovary's outer surface and are the most common, up to 90% of all cases [161]. The symptoms present are not characteristic of the disease, such as disturbing sensations of abdominal distension, periodic pains in the groin, problems with swallowing and eating, vomiting, and frequent urination [162]. It is possible to confuse this symptomatology with gastrointestinal diseases, bloating, and premenstrual syndrome, which can lead to additional difficulties in early diagnosis [163]. In their study, Wang et al. identified that battalions of immune cell abundance had a lower level in higher CTC.246B18.8 expression groups. In addition, a high CTC.246B18.8 expression was related to concerns for the lymphocyte level. LncRNAs identified as prognosis-associated, including RP5–1076A10.1, TM4SF1-AS1, RP11–409C1.2, AC020916.1, RP11–608G10.3, and CTC.246B18.8. The higher expression of the eighth RNA increased the possibility of bad results; there was also a change in the immune profile [164].

Paclitaxel, also known and marketed under the brand name Taxol, is a chemotherapy medication from the Pacific yew tree [165]. It is extensively used against different types of cancer, such as ovarian, breast, lung, and pancreatic cancers, and Kaposi’s sarcoma [166]. Gefitinib, also marketed as Iressa, is a targeted therapy mostly used against non-small cell lung cancer [167]. As a tyrosine kinase inhibitor, it is developed to affect the function of the epidermal growth factor receptor [168]. Li et al. analyzed cuproptosis-related lncRNAs in ovarian cancer to determine the molecular subtypes and develop a prognostic signature. Their results showed different links to prognosis, immunity, and drug reactions for the top 10 lncRNAs. The identified subtypes demonstrate the heterogeneity of ovarian cancer and the possible biomarkers of prognosis and drug response [169]. Characterizing immune profiles in the context of assessing immune system function and guiding the development of new immunotherapy strategies has relevance to several diseases, such as various types of cancer and autoimmune diseases [170]. Liu et al. developed a prognostic signature of four cuproptosis-related lncRNAs in ovarian cancer. They identified that the signature effectively separated patients with ovarian cancer into high- and low-risk subgroups, which was significantly associated with overall survival, immune profiles and drug sensitivity. Their approach introduces a new perspective with respect to individualized treatment strategies for ovarian cancer [171]. High tumor mutational burden (TMB) is often associated with an improved response to immunotherapy as more mutations could potentially generate novel antigens; as such, high TMB level might serve as a potential predictive marker of how cancer patients might respond to a certain type of treatment [172]. In a study by Guo et al., a prognostic model of ovarian serous cystadenocarcinoma was built by constructing cuproptosis-related lncRNAs. The results of the study showed that they identified 5 lncRNAs that were significant. The cross-validation has proven to have a strong predictive effect on patients’ prognosis and has a significant correlation with immune cell infiltration and TMB. Overall, the model provides information that can help patients better understand their outcomes and think about the appropriate treatment strategy [85].

### LncRNAs in cuproptosis-mediated gastric *cancer*

Gastric cancer, or stomach cancer, is a malignant tumor originating from the gastric mucosa [173]. Gastric cancer is a matter of global health concern, with high incidence rates in East Asia, Eastern and Western Europe, and parts of South America [174]. Gastric cancer is generally asymptomatic in the early stages, as the symptoms may be vague and nonspecific [175]. Most patients are diagnosed in the advanced stages due to the absence of screening and the limited sensitivity of common endoscopic techniques [176]. The prognosis of gastric cancer varies depending on the stage at diagnosis [177]. The major factor affecting the prognosis of gastric cancer is the extent of the spread of the disease and whether the target organ has been invaded [178]. Early gastric cancer is curable with complete surgical resection and offers behind excellent 5-year survival rates [179]. However, advanced-stage cancer, which has a propensity for early metastasis, has dismal 5-year overall survival values for most current treatments [180]. The 5-year survival of advanced gastric cancer is still low, with a rate of no more than 5%-20%, emphasizing the need for early detection and prevention [181]. Zhao et al. constructed a prediction model based on cuproptosis-related gene expression levels to evaluate gastric cancer's clinical prognosis and immunotherapy efficacy. It concluded that high-risk patients had adverse overall survival, the risk score was an independent prognostic predictor, and a connection was established between the risk score and immune cell infiltration [182].

Cholesterol metabolism involves synthesis, absorption, transport, and excretion [183]. It is essential to cell membrane structure, hormone, and bile acid production [184]. Imbalance could cause cardiovascular disease [185]. The liver, through lipoprotein particles, such as low-density lipoprotein and high-density lipoprotein, controls the body's cholesterol level [186]. Feng et al. conducted a study on a novel nomogram based on cuproptosis-related lncRNA to improve prognosis prediction in gastric cancer. They discovered that the nomogram based on cuproptosis-related lncRNAs was effective, as patients in the high-risk group using this nomogram to predict outcome had a poorer prognosis than those in the low-risk group. This model was better than clinicopathological parameters in predicting outcomes, and it was found to be associated with cholesterol metabolism and immune function. The correlation between CD209 and HAVCR2 immune checkpoints and risk scores was validated in the experiment [187]. The oncogenes are an example of a gene mutation that may cause cancer development [188]. A somatic mutation occurs in non-germline cells after birth and is not inherited [189, 190]. Somatic mutations cause cancer or other diseases when they functionally affect cell division or function and are consigned due to environmental factors or other cellular errors [191]. Yin et al. developed and assessed a novel cuproptosis-related lncRNA signature to predict the prognosis of gastric cancer. The authors built a risk prediction model according to cuproptosis-related lncRNAs and proved that it could effectively classify patients into high- and low-risk groups. The model showed high prediction ability and was allied with the alterations in immune function, somatic mutation, and drug sensitivity [192].

Natural drug sensitivity refers to the inherent responsiveness of organisms or cells to drugs obtained from natural sources, such as plants, fungi, or microorganisms [193]. This sensitivity depends on various genetic, biochemical, and physiological factors and participates in the drug efficacy and development of approaches to personalized therapy [194]. Song et al. created a metal-dependent programmed cell death-related lncRNA prognostic signature for gastric cancer and assessed natural drug sensitivity. The 12-lncRNA signature model indicated the worse prognosis of high-risk patients and effectively predicted the natural antitumor drug sensitivity [195]. The tumor microenvironment can be defined as the cellular and extracellular composite surrounding a tumor [196]. It comprises immune cells, fibroblasts, blood vessels, and extracellular matrix [197]. The components in the tumor microenvironment may result in tumor growth and metastasis and affect a tumor's responsiveness to treatment [198]. Therefore, it is a critical area of interest for researchers who strive to understand and treat cancer [199, 200]. Huang et al. conducted a study investigating the diverse tumor microenvironment landscapes in gastric cancer using cuproptosis-related lncRNAs. The researchers developed a six-lncRNA signature that predicts patient survival and displays differences in immune activation and TME characteristics. Their study concludes that high-risk subgroups could have higher TME scores and receive better anti-PD-1 immune checkpoint blockade [201, 202].

Generally, antineoplastic drugs are defined as a broad group of medicines used to fight the development and spread of cancer [203]. Their main effect is achieved by targeting and killing abnormal and rapidly dividing cancer cells [204]. It is also important to note that there are several classes of these drugs, including alkylating agents, antimetabolites, and taxanes, among others, with all of them ultimately aimed at inhibiting tumor growth and spread [205]. It also follows from the definition that these drugs may negatively affect normal cells, causing a range of side effects [206, 207]. Tu et al. generated a cuproptosis-related lncRNA gene signature to predict the prognosis of gastric adenocarcinoma and assess the effect of antineoplastic drugs. In particular, the study has identified a six-lncRNA signature that correlated with patient survival, with high-risk patients demonstrating superior immunotherapy responses and different sensitivities to specific drugs [208]. Another study by Wang et al. developed a novel risk model by cuproptosis-related long non-coding RNAs for gastric cancer and analyzed the immune landscape. They identified 10 CRLs for predicting prognosis and their relevance with risk scores with immune cell infiltration, drug sensitivity, and tumor mutation burden. This model showed the potential to predict patient prognosis and guide immunotherapy [209, 210].

### LncRNAs in cuproptosis-mediated pancreatic *cancer*

Pancreatic cancer is an aggressive form of cancer that starts in the tissues of the pancreas, an organ lying behind the lower part of the stomach [211]. The pancreas helps in digestion and the regulation of blood sugar through exocrine and endocrine functions [212, 213]. Most carcinomas of the pancreas start in the exocrine cells that produce digestive enzymes [214]. The most common type of pancreatic cancer is pancreatic ductal adenocarcinoma, constituting over 90% of all pancreatic carcinomas [215]. The prognosis of pancreatic cancer remains poor, having more than 10% five-year survival rate due to reasons including late diagnosis and the aggressiveness of the disease [216]. Despite this, pancreatic cancer is studied more and more extensively as continually accumulating science is unveiling more molecular mechanisms of the disease and new diagnostic markers and targets for therapy [217, 218].

Moreover, thanks to the advances in imaging technologies and genetic testing, personalized care can be adopted for better and more timely treatment [219]. CASC8, also known as CAN8 or Cancer Susceptibility 8, is a long non-coding RNA lncRNA associated with the susceptibility to, occurrence, and progress of this type of disease [220, 221]. It influences the process of gene expression and the development of a tumor [222]. As a lncRNA associated with cancer, its expression also correlates with other carcinomas, making the CASC8 a biomarker in blood and tumor tissue [223, 224]. Likewise, therapeutic agents can be developed to influence its expression [225]. A study by Yao et al. developed a signature of five lncRNAs associated with cuproptosis and affecting patients’ outcomes and the immune environment of the tumor in pancreatic cancer, retrieved from TCGA and ICGC cohorts. CASC8 was pointed out in the study as a five-gene signature influencing both patients’ outcomes and immune environment as having a higher expression and correlating to a worse outcome [226]. Similar research was conducted by Jiang et al. to construct a predictive model based on the cuproptosis-related lncRNAs in the case of pancreatic cancer. The researchers have found that 181 lncRNAs are relevant for the case in question and have identified the five specific lncRNA risk models. Overall, the model can be considered effective as it led to the difference in prognosis of the patients in the high- and low-risk groups [227, 228]. Sun et al. have constructed the predictive model by using the single-cell analysis of cuproptosis-related lncRNAs and their impact on the immune microenvironment in pancreatic cancer. The CIR-score, which integrates the factors of lncRNA signatures and clinical traits, could predict the prognosis, thus informing individual immunotherapy [229].

Glycolysis is a metabolic pathway that converts glucose to pyruvate while generating ATP and NADH [230]. Glycolysis is essential for energy production and metabolism, both anaerobically and aerobically, and it is also a precursor for many biosynthetic pathways [231]. Pancreatic adenocarcinoma’s cuproptosis-related lncRNA was studied through systemic analysis by Chen et al., focusing on the molecular mechanism of LINC00853. The study also developed a prognostic signature based on four lncRNAs and analyzed LINC00853’s role in glycolysis and cell proliferation [232, 233].

Tumor metabolism is a term used to describe the metabolic processes altered within cancer cells to promote their rapid growth, survival, and proliferation [234]. The defining feature of cancer cells is the presence of altered metabolic characteristics, distinct from those of normal cells, allowing them to out-compete normal cells in the harsh and often nutrient-poor conditions of tumors [235, 236]. For example, many cancer cells display increased glucose uptake and glycolysis rates. This metabolic characteristic has been used diagnostically in positron emission tomography by tagging a radiolabeled glucose analogue [237]. Upregulation of glucose transporters, such as GLUT1, and the key glycolytic enzymes, such as hexokinase 2, fuel the high glycolytic flux characteristic of many tumors [238]. The end product of glycolysis in cancer cells is lactate, which is perfused into the local microenvironment, contributing to the acidosis of the tumor microenvironment, which can promote cancer cell invasion and metastasis [239, 240]. Wang et al. studied the relationship between cuproptosis-related lncRNAs and the metabolism of tumors and the immune microenvironment and pancreatic cancer. They constructed a predictive model in their analysis, which determined that higher risk scores correlated with a worse cancer survival prognosis and a more immunosuppressive panorama in pancreatic cancer [241]. A study by Chen et al. developed a prognostic signature built on the cuproptosis-related lncRNAs and discovered its association with the prognosis of pancreatic cancer and tumor microenvironment. The study highlights the importance of the signature as a potential biomarker for prognosis and effect on drug targets [242].

### LncRNAs in cuproptosis-mediated liver *cancer*

**Fig. 4:**
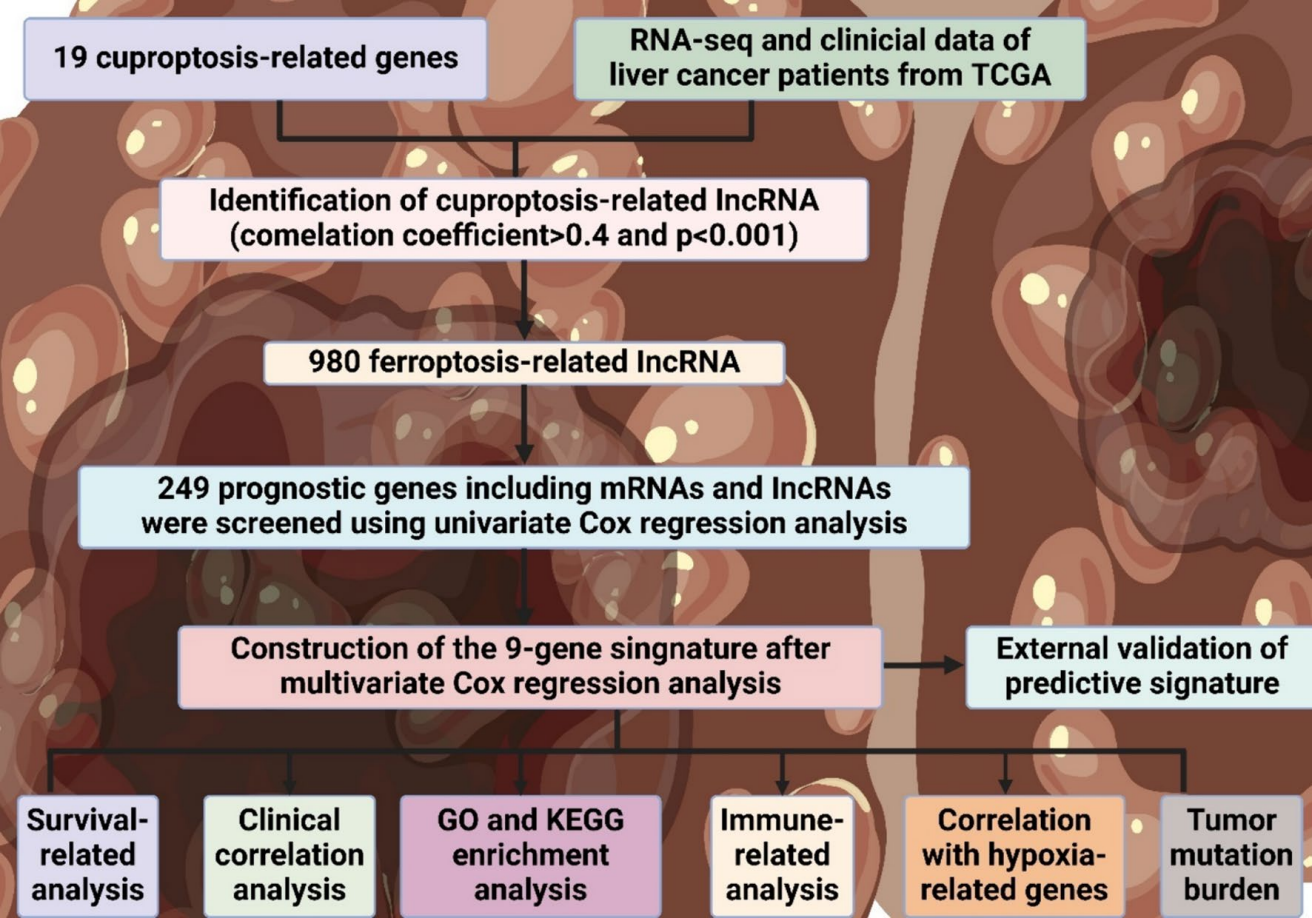
The figure details the identification of 9-gene prognostic signature in liver cancer from TCGA data, using cuproptosis and ferroptosis-related lncRNAs, with analyses including survival, clinical correlation, GO/KEGG, immune, hypoxia, and tumor mutation burden

HCC accounts for roughly 75–85% of all liver cancer cases [243]. The prognosis for liver cancer is highly varied, dependent on how advanced the cancer is and the relative health of the patient [244]. Early liver cancer that is normally surgically removed or treated with transplantation has a relatively good prognosis [245]. The five-year survival rates are elevated, bridging 30–50% [246]. For advanced liver cancer patients, the prognosis is less good, with 5-year survival rates roughly under 20% [247]. Liu et al. developed and validated a predictive model for liver cancer based on cuproptosis-related mRNAs and lncRNAs. Five-year survival performances evaluate the prediction capacity of our model. With the model, other features, including the estimations of immune cell infiltration, TMB, and antitumor drug sensitivity, are also useful for clinical applications, as shown in Fig. 4 [248].

CYTOR is a novel, non-coding RNA regulating cell growth and proliferation [249]. It behaves as a molecular scaffold, which exerts a widespread effect on the signaling pathways and expression of the genes [250]. CYTOR’s dysregulation has been observed in cancer, creating an excellent peculiarity of a potential therapeutic target [251]. Chen et al. constructed a prognostic signature based on the cuproptosis-related lncRNAs in HCC, such as CYTOR, LINC00205, and LINC01184. The signature demonstrated an excellent perspective in forecasting the prognosis and the response to immunotherapy by the patient, which has been verified by both internal assessment and in vitro studies [252]. HCC is the most common type of liver cancer and develops in the context of chronic liver disease such as cirrhosis or hepatitis [253]. It is characterized by fast growth, poor prognosis, and resistance to therapy, so early detection and timely treatment are essential for the survival of patients [254, 255]. Wang et al. present a cuproptosis-related lncRNA risk model for HCC and identifies nine prognostic lncRNAs. When this model is applied, it is possible to precisely predict patients’ prognosis, their sensitivity to immunotherapy, and the condition of the immune microenvironment of their tumor. All this indicates that it is of high clinical value to predict which patients are likely to respond positively to immunotherapy [256]. Evaluating chemotherapy effectiveness means assessing whether the treatment has succeeded, which can be determined through a range of clinical outcomes, such as tumor shrinkage, progression-free survival, and overall survival [257]. Imaging studies, biomarker analysis, and patient-reported outcomes are the techniques for assessing these results [258]. Such evaluation can help decide improvement made in the regimen, while the outcomes are used to monitor patient care [259]. Liu et al. developed a predictive model for HCC with eight lncRNAs associated with cuproptosis.

Along with predicting patient outcomes, the model can be used to evaluate chemotherapy effectiveness; the differences found in immune functions and drug sensitivity between high- and low-risk groups are significant [260]. Non-apoptotic therapeutics target processes not specifically involved in the induction of apoptosis [261]. These alternatives include actions related to necroptosis, autophagy, and pyroptosis, among other mechanisms [42]. Applying these therapies offers new approaches to modulating diseases and providing additional options for apoptotic pathways [262]. Non-apoptotic therapeutics are viable in various cases and bring greater advances in treatment methods [263]. Zhang et al. developed a cuproptosis-related lncRNA signature for hepatocellular carcinoma to predict patients’ overall survival and response to immunotherapy. Different analyses validated the examination and provided a new perspective on HCC treatment not based on apoptosis [264] (Fig. 4; Table 1).

**Table 1:** This table summarizes various studies on cuproptosis-related lncRNAs, highlighting their impact on prognosis, therapy response, and immune microenvironment in different cancers, along with key findings and significant lncRNAs

Study Focus	Cancer Type	Key Findings	Significant lncRNAs	Additional Insights	Reference
Cuproptosis-related gene in breast cancer	Breast Cancer	SLC31A1 impacts prognosis, chemosensitivity	LINC01614/miR-204-5p/SLC31A1	Immune cell infiltration	[111]
A predictive model with cuproptosis lncRNAs	Breast Cancer	Predicts therapy response, survival	Six key lncRNAs	Risk classification	[116]
Prognostic model, immune microenvironment	Breast Cancer	High-risk: lower survival, mutations	11 lncRNAs	Mutation profiles	[120]
2-lncRNA signature for prognosis	Breast Cancer	BCCuS predicts therapy response	2-lncRNA signature	Prognostic factor	[121]
Prognostic, immune microenvironment model	Breast Cancer	10 lncRNAs predict survival, sensitivity	10 lncRNAs	Drug sensitivity	[122]
Prognostic, immunotherapeutic value	Lung Cancer (LUAD)	16 lncRNAs predict survival, immune escape	16 lncRNAs	Immunotherapy potential	[265]
Prognostic signature post-radiation therapy	NSCLC	Predicts patient prognosis	Six lncRNAs	Radiation response	[266]
Prognosis, immunotherapy responses	LUAD	Six lncRNAs predict therapy response	Six lncRNAs	Immunotherapy strategies	[142]
Prognostic signature, immune landscape	LUAD	Predicts survival, drug sensitivity	Ten lncRNAs	GSVA and GSEA analyses	[4]
Predictive signature for immunotherapy	LUAD	Guides individualized treatment	Six lncRNAs	Experimental validation	[150]
Multi-omics approach in ovarian cancer	Ovarian Cancer	Six lncRNAs linked to prognosis	CTC.246B18.8	Immune response	[164]
Molecular subtypes, prognostic signature	Ovarian Cancer	Ten lncRNAs linked to prognosis	Ten lncRNAs	Heterogeneity	[169]
Prognostic signature in ovarian cancer	Ovarian Cancer	Stratifies survival, drug sensitivity	Four lncRNAs	Individualized treatment	[171]
Prognostic model in serous cystadenocarcinoma	Ovarian Cancer	Predicts outcomes, immune infiltration	Five lncRNAs	Tumor mutational burden	[85]
A predictive model for gastric cancer	Gastric Cancer	High-risk: poorer survival	Cuproptosis-related genes	Immunotherapy efficacy	[182]
Cuproptosis-related lncRNA nomogram	Gastric Cancer	Improves prognosis prediction	Cuproptosis-related lncRNAs	Cholesterol metabolism	[187]
Novel lncRNA signature for prognosis	Gastric Cancer	Predicts risk, immune function	Cuproptosis-related lncRNAs	Somatic mutation	[192]
lncRNA prognostic signature	Gastric Cancer	Predicts drug sensitivity	12-lncRNA signature	Natural drug sensitivity	[195]
TME landscapes in gastric cancer	Gastric Cancer	Six-lncRNA predicts survival	Six-lncRNA signature	Immunotherapy responses	[201]
lncRNA signature for prognosis	Gastric Cancer	Predicts drug sensitivity	Six-lncRNA signature	Antineoplastic drugs	[208]
Novel risk model based on CRLs	Gastric Cancer	Predicts prognosis, guides immunotherapy	10 lncRNAs	Tumor mutation burden	[209]
lncRNA signature for pancreatic cancer	Pancreatic Cancer	Five-lncRNA predicts outcomes	Five lncRNAs	Immune response	[226]
Prognostic model in pancreatic cancer	Pancreatic Cancer	Five-lncRNA predicts prognosis	Five lncRNAs	Risk stratification	[227]
CIR-score and immune environment	Pancreatic Cancer	Guides immunotherapy	Cuproptosis-related lncRNAs	Individualized therapy	[229]
Molecular mechanism of LINC00853	Pancreatic Cancer	Role in glycolysis, proliferation	Four lncRNAs	Cell proliferation	[232]
lncRNAs and tumor metabolism	Pancreatic Cancer	Links risk to outcomes	Cuproptosis-related lncRNAs	Immune microenvironment	[241]
Prognostic signature, tumor environment	Pancreatic Cancer	Biomarker potential	Cuproptosis-related lncRNAs	Therapeutic targeting	[242]
A predictive model for liver cancer	Liver Cancer	Predicts survival, drug sensitivity	Cuproptosis-related lncRNAs	Immune cell infiltration	[248]
Prognostic signature in HCC	Liver Cancer	Predicts prognosis, immunotherapy	CYTOR, LINC00205, LINC01184	Immunotherapy response	[252]
lncRNA risk model for HCC	Liver Cancer	Nine lncRNAs predict prognosis	Nine lncRNAs	Tumor environment	[256]
Prognostic model, chemotherapy effectiveness	Liver Cancer	Eight-lncRNA predicts outcomes	Eight lncRNAs	Drug sensitivity	[260]
Cuproptosis-related lncRNA signature for HCC	Liver Cancer	Predicts prognosis, immunotherapy	Cuproptosis-related lncRNAs	Non-apoptotic strategies	[264]

## Tumor heterogeneity and lncRNAs in cuproptosis

The expression and functionality of lncRNAs may be strongly impacted by this variability, which makes it more difficult to identify universal diagnostics or effective treatment approaches for cancer [267]. Due to genetic and epigenetic variations, many cancer types often have unique lncRNA expression patterns. For instance, lung cancer has high expression levels of MALAT1, which encourages metastasis and adds to treatment resistance [268]. On the other hand, MALAT1 in liver cancer has been linked to the promotion of tumor development via a variety of mechanisms, including angiogenesis and cell cycle modulation [269]. The difficulty of using MALAT1 as a universal biomarker for cuproptosis is highlighted by the variation in its function across various malignancies [270]. While MALAT1 may have a less direct role in this cell death pathway in liver cancer, it may affect lung cancer patients' responsiveness to medicines that cause cuproptosis by controlling genes associated with mitochondrial activity [271].

Different parts of a single tumor may express different amounts of lncRNA because of interactions with immune cells, hypoxia, or the availability of nutrients [272]. HOTAIR is elevated in some tumor areas in breast cancer, especially those with high hypoxia levels. HOTAIR can alter gene expression and chromatin remodeling, which varies the tumor reaction to treatment [273]. Because of improved survival signaling pathways, tumor areas with high HOTAIR expression may be more resistant to cuproptosis-inducing drugs. In contrast, tumor regions with low HOTAIR expression may be more vulnerable to such treatments. Because of this intra-tumoral heterogeneity, HOTAIR is not a reliable predictive diagnostic for the whole tumor [274].

The extracellular matrix, fibroblasts, and immune cells that make up the TME are important factors in the expression of lncRNA [275]. The TME affects the lncRNA PVT1 in pancreatic cancer, especially in regions with a high stromal composition. It has been shown that PVT1 interacts with TME elements to support cancer cell survival and apoptosis resistance [276]. PVT1 may influence the oxidative stress response and copper ion homeostasis in the setting of cuproptosis, increasing the resistance of certain tumor areas to cell death [277]. Rather than depending only on single lncRNAs for prognosis, biomarker panels must be used due to the variability in lncRNA expression [278]. LncRNAs, including MALAT1, H19, and NEAT1, have been suggested to predict patient outcomes in lung cancer [268] more precisely. The combined study of these lncRNAs may provide a more thorough knowledge of the behavior of the tumor, including its response to cuproptosis-inducing treatments. These lncRNAs are engaged in several aspects of tumor biology, including proliferation, metastasis, and cell death control [279].

## Clinical relevance of lncRNAs in cuproptosis

LncRNAs have the potential to be predictive biomarkers in cuproptosis-mediated cancer treatment; nevertheless, in order to ensure practical application, extensive validation is necessary [280]. Because cancer is a very varied disease, biomarker validation is a multi-step procedure that involves determining the sensitivity, specificity, and repeatability of lncRNAs across a range of patient populations [281]. Determining the sensitivity and specificity of lncRNAs and their capacity to identify people with the illness and those without it accurately is essential to using them as trustworthy biomarkers [282]. The PVT1, which has been linked to several malignancies, has shown potential as a biomarker for prognosticating patients and therapeutic response. Its expression levels, however, may change dramatically across cancer types and even among people who have the same cancer type. Therefore, it's crucial to set a constant threshold for PVT1 expression that reliably forecasts cuproptosis-related events [283].

Another important component in the validation of biomarkers is reproducibility. In breast and colorectal malignancies, the lncRNA HOTAIR has been thoroughly investigated and has been linked to a poor prognosis as well as treatment resistance [284]. HOTAIR must, however, show consistent predictive efficacy across many cohorts, including individuals with various genetic backgrounds and clinical circumstances, in order to be validated as a biomarker for cuproptosis [281]. Clinical validation includes large-scale research to assess the biomarker's usefulness in real-world scenarios after its original discovery and validation in preclinical investigations. For example, the lncRNA MALAT1 has been investigated as a lung cancer prognostic marker [285].

## Data integration

To better understand the involvement of lncRNAs in cuproptosis, a recently identified kind of programmed cell death connected to copper metabolism, it is imperative to integrate multiple datasets. Through the integration of genetic, transcriptome, proteome, and clinical data, scientists may reveal complex connections between lncRNAs and cuproptosis, which might provide novel treatment opportunities [81]. The GAE-LGA model uses graph autoencoders to integrate multi-omics data and find relationships between lncRNAs and protein-coding genes (PCGs). The model successfully predicts lncRNA-PCG connections by capturing functional similarities and cross-omics correlations. Using this method, scientists may investigate how lncRNAs might interfere with PCG activities and affect cuproptosis pathways. The incorporation of multi-omics characteristics considerably enhances the resilience of these forecasts, offering an effective instrument for comprehending the regulatory functions of lncRNAs in cuproptosis [286].

Cuproptosis-related lncRNAs in lung adenocarcinoma (LUAD) were identified by combining gene expression, clinical outcomes, and mutation data from The Cancer Genome Atlas (TCGA). Through the use of both univariate and multivariate Cox regression analysis, a predictive model including 15 lncRNAs linked to cuproptosis was created. This model demonstrates the potential of integrated data to aid clinical decision-making and treatment options since it not only predicted patient survival but also offered insights into immune escape pathways and medication sensitivity [287]. TCGA data were used in research on gastric cancer to create a nomogram based on lncRNAs associated with cuproptosis. Key lncRNAs that may function as prognostic indicators were found by the researchers by combining gene expression patterns with clinical and survival data. Kaplan–Meier survival analysis was used to evaluate the model's efficacy and show how integrated techniques might improve knowledge of lncRNA contributions to cancer development and patient outcomes [187].

## Challenges

LncRNAs are exceedingly difficult to target therapeutically because of their complicated structures and the need for very specialized delivery methods to prevent off-target effects [288]. Since lncRNAs do not encode proteins, their modes of action often include complex interactions with proteins, RNA, and DNA that may vary greatly based on the biological setting [1]. Because of this intricacy, specific techniques for lncRNA regulation must be developed, especially when it comes to cuproptosis, where aberrant lncRNA activity may either promote or prevent cell death [289]. Secondary structures, including hairpins, loops, and pseudoknots, are often seen in lncRNAs and are essential to their function. It isn't easy to create compounds that can selectively target lncRNAs without interfering with other RNA species because of these structural characteristics [290]. Many strategies are being investigated to modify lncRNA activity, including CRISPR/Cas9-based technologies, siRNAs, and antisense oligonucleotides (ASOs) [291]. ASOs may be made to attach to certain sequences in lncRNAs, causing them to degrade or preventing them from interacting with target molecules. However, the stability of the lncRNA-ASO complexes in the cellular environment and the accessibility of the target sequences determine how successful these techniques are [292].

Another significant obstacle is the delivery of medicinal drugs that target lncRNAs. In order to minimize off-target effects, efficient delivery methods must guarantee that the therapeutic molecules reach the targeted cells and tissues in adequate amounts [293]. To improve the effectiveness and selectivity of lncRNA-targeted treatments, lipid-based carriers, viral vectors, and nanoparticle-based delivery methods are being developed [293]. Lipid nanoparticles have shown effective in delivering siRNAs that target the long non-coding RNA MALAT1 in preclinical models of lung cancer. Since lncRNAs may work in a variety of tissues and biological pathways, the possibility of off-target consequences is still a serious worry [293].

A thorough grasp of lncRNA function in cuproptosis and other cancer-related pathways is necessary in order to integrate them into precision medicine approaches. Personalized techniques that modify therapy according to the unique long non-coding RNA expression patterns of a patient's tumor [294]. Tailored medicines may be created to block the action of the lncRNA HOTAIR, which is increased in malignancies and linked to a bad prognosis. This would make the tumor more susceptible to cuproptosis. Nevertheless, more testing is necessary to guarantee that focusing on HOTAIR won't negatively impact regular cellular processes [295].

## Conclusion

The exploration of lncRNAs can be used as prognostic markers and therapeutic targets in cuproptosis-mediated cancer; it presents a promising way to develop cancer diagnostics and treatment. Cuproptosis is a newly identified form of programmed cell death regulated by copper ions and distinguished from apoptosis, necroptosis, and ferroptosis, which are induced by similar biochemical pathways but regulated by glucose restriction. The findings from various research studies emphasize the significant involvement of lncRNAs in modulating key pathways associated with cuproptosis across different cancer types, including breast, lung, ovarian, gastric, and pancreatic cancers. Our analysis reveals that specific lncRNAs, such as MALAT1, HOTAIR, and PVT1, interact with crucial proteins and pathways that govern mitochondrial function and oxidative stress responses, thereby influencing the susceptibility of cancer cells to cuproptosis. The identification of these lncRNAs as potential biomarkers opens new avenues for predicting cancer prognosis and therapy response. For instance, the SLC31A1-related axis in breast cancer and the role of specific lncRNAs in LAUD have shown promising results in predicting patient outcomes and guiding therapeutic strategies.

Moreover, several studies have developed predictive models based on cuproptosis-related lncRNAs, showing their potential to predict patient outcomes, therapy sensitivity, and immune response. These models, such as those applied in lung adenocarcinoma and hepatocellular carcinoma, underscore the clinical relevance of lncRNAs in personalized cancer treatment. Furthermore, the study underscores the potential of leveraging these lncRNAs as therapeutic targets to induce cuproptosis selectively in cancer cells, offering a novel approach to overcome resistance to conventional therapies. The development of predictive models based on cuproptosis-related lncRNAs, as seen in ovarian and pancreatic cancers, further supports the clinical relevance of these molecules in personalizing cancer treatment.

## Future perspectives

### Emerging research directions

Emerging research direction in cuproptosis and lncRNAs emerges and can revolutionize our understanding and treatment of diseases, mostly cancer [296]. Mechanistic insights in cuproptosis have been a focus of investigations. However, on the one hand, the interests of scientists are directed at understanding how mitochondrial failure and cell death are triggered by the accumulation of copper [297]. Several investigations have been conducted to understand molecular interactions and signaling pathways of copper, mitochondrial proteins, and events in cuproptosis [298]. On the other hand, intensive attention is paid to understanding how the processes of copper metabolism and cuproptosis are regulated by lncRNAs [299]. There are lncRNAs capable of controlling the expression of copper transfer systems, including CTR1 ATP7A and ATP7B, responsible for the uptake and efflux of copper [300]. The assumption is that these carriers and modulators contribute to increased intracellular copper levels and cuproptosis susceptibility [301]. Moreover, the production of COUP-TFII lncRNA is considered to govern mitochondrial-driven stress response and, thus, copper-induced damage [302].

Therapeutic research has not been left behind, and an emerging research direction is the cultivation of novel treatments modulating cuproptosis. The efforts are directed to damage cancer cells [299] specifically. As a result, several copper-modulating agents catching scientists' attention are created [303]. Copper chelators are produced for intracellular copper level reduction [304]. Some drugs induce cuproptosis only; these novel drugs kill cancer cells and contribute to resistance overcoming [305]. An emerging research direction of biomarker discovery is to identify cuproptosis-associated expression profiles of lncRNA [306]. This may lead to novel methods of cancer diagnosis, early cancer identification, and monitoring of the disease process to administer the best allergy treatment. Individualized treatment strategies may also be formed as a result [307].

### Potential clinical applications

The investigation into cuproptosis and its regulation by lncRNAs presents valuable clinical applications, particularly in cancer treatment [308]. One of the primary target treatment options revolves around investigating and utilizing the mechanism through which cuproptosis kills cancerous cells rather than just healthy ones [309]. Copper-induced cell death is a potentially promising treatment avenue due to the improved susceptibility of cancer cells to this particular symptom [310]. Scientists are developing both cuproptosis-inducing drugs and medications that alter the metabolism of copper as a potential approach to treatment [311]. For instance, copper chelators could affect the intracellular levels of copper [312]. While present in healthy cells, the compounds that only kill tumor cells could also revolutionize the treatment industry, rescuing many patients who are resistant to existing therapeutic processes [313].

Similarly, cuproptosis-related and lncRNA monitoring biomarkers can significantly increase the accuracy of cancer diagnostics and overall prognosis [314]. This mechanism can allow scientists to find cancer solutions within a timeframe starting earlier than the first symptoms and until the imminent danger of metastasis [315]. The monitoring processes can assess the individual treatment plans for effectiveness against a particular type of cancer and plan new product development and testing accordingly [316]. Combining different therapy types based on the conference material, such as chemotherapy, immunotherapy, or targeted therapy in conjunction with cuproptosis-inducing agents, can also improve the overall practical effectiveness of these treatments and counter-resistance [317].

## Data Availability

No datasets were generated or analyzed during the current study.
